# Children's adherence to health behavior recommendations associated with reducing risk of non-communicable disease

**DOI:** 10.1016/j.pmedr.2017.10.006

**Published:** 2017-10-10

**Authors:** Louise L. Hardy, Seema Mihrshahi, William Bellew, Adrian Bauman, Ding Ding

**Affiliations:** Prevention Research Collaboration, Sydney School of Public Health, University of Sydney, NSW, Australia

**Keywords:** Risk factors, Diet, Physical activity, Oral health, Adolescent, Screen time

## Abstract

The aim of this study is to report the proportions of Australian children age 5–16 years meeting six health behavior recommendations associated with reducing risk of non-communicable disease. Data comes from a representative cross-sectional population survey conducted in 2015. Parents completed a health behavior questionnaire for children age < 10 years and adolescents age > 10 years self-reported. Adherence rates were calculated separately for children and adolescents on meeting recommendations for fruit (2-serves/day), vegetables (5-serves/day), physical activity (≥ 60 min/day), screen-time (< 2 h/day), oral health (brush-teeth twice daily) and sleep (children 9–11 h/night; adolescents: 8–10 h/night). Participants were 3884 children and 3671 adolescents. Adherence to recommendations was low, with children adhering to an average of 2.5 and adolescents to 2.3 of six recommendations. Overall, recommendation adherence rates were 7% for vegetables, 18% for screen-time, 20% for physical activity, 56% for sleep, 67% for dental (teeth brushing) 79% for fruit; 3.3% reported zero adherence with recommendations and < 0.5% adhered to all six recommendations. There was evidence of social disparity in adherence rates; children and adolescents from low socioeconomic neighborhoods met fewer recommendations and were less likely to meet screen-time and dental recommendations, compared with high socioeconomic peers. Children and adolescents from rural areas met more recommendations, compared with urban peers. Children's and adolescents' adherence to health behavior recommendations is sub-optimal, exposing them to risk of developing non-communicable diseases during adulthood. Better communication and health promotion strategies are required to improve parents' and children's awareness of and adherence to health behavior recommendations.

## Highlights

• < 0.5% of 5–16 year old children adhered to all 6 health behavior recommendations.
• 3.3% of 5–16 year old children adhered no health behavior recommendation.
• Low adherence places children at increased risk of non-communicable disease (NCDs).
• Increased rates of NCDs may have future health and economic implications.

## 1 Introduction

Globally, the increase in the prevalence of non-communicable diseases (NCDs) has coincided with the increase in populations adopting unhealthy lifestyle behaviors (Swinburn et al., 2011). Changes in the social, physical, and economic environment are key drivers of risk factors for NCDs (Swinburn et al., 2011, Forouzanfar et al., 2015). The development of health recommendations is one public health strategy to assist populations reduce their lifestyle risk related NCDs and improve health and well-being. Monitoring population compliance with health recommendations is an important aspect of public health planning.

The foundations for many lifestyle behaviors begin during childhood yet in many countries children's adherence to multiple health behavior recommendations is generally low (Kovacs et al., 2014), and little is known about the barriers that contribute to poor adherence in children. In adults, sociodemographic factors may be associated with adherence to health behavior recommendations (Forouzanfar et al., 2015, Ding et al., 2015). Potential reasons for children's intentional or unintentional non-adherence to health behavior recommendations may include children and parents' lack of awareness, misunderstanding, inadequate knowledge of the consequences of non-adherence, recommendations being too complex, high financial cost and lack of perceived immediate health benefits.

Determining the prevalence of adherence to recommendations, and the sociodemographic characteristics of children with low adherence could be useful to guide the development and targeting of health-promotion interventions. We use population health surveillance data collected on a representative sample of children age 5–16 years to describe the proportion of children who 1) meet the Australian recommendations for fruit and vegetable intake, physical activity, screen-time, sleep, and teeth brushing, 2) who are aware of health recommendations and, 3) identify the sociodemographic characteristics of children who are less likely to adherence to these recommendations.

## 2 Methods

We use data from the 2015 Schools Physical Activity and Nutrition Survey, a serial representative cross-sectional population health surveillance survey of children age 5–16 years living in New South Wales (NSW), Australia. A detailed description of the survey methodology is published elsewhere (Hardy et al., 2017). Briefly, the surveys are designed to be representative of school age children in terms of type of school, residence, and socioeconomic status. The sample size was based on detecting a 10% differences between geographically defined sub-groups of children with 80% power and an alpha level of 0.05. The study protocols are comparable for each survey year and data are collected by trained field teams during February to April. Informed consent from each child's parent/guardian was a requirement for participation. Ethics approvals were granted by the University of Sydney, the NSW Department of Education and Training and the NSW Catholic Education Commission.

### 2.1 Measures

Table 1 summarizes the recommendations for children and adolescent's daily fruit and vegetable intake, physical activity (PA), recreational screen-time, sleep, and oral hygiene. Validated questions were used to collect information on these health behaviors to determine whether children were meeting the recommendations. Daily intake of fruit and vegetables was collected using a validated short food frequency questionnaire developed for population-based monitoring surveys (Flood et al., 2005), teeth-brushing was assessed using validated questions from a national population oral health survey (Hirshkowitz et al., 2015), sleep duration was collected using questions adapted from validated sleeping habits and hygiene questionnaires (Wolfson et al., 2003), PA levels were determined using a validated single item screening measure of moderate-to-vigorous physical activity (Prochaska et al., 2001), and recreational screen time (television (TV), videos/DVDs, computer, smart phone, tablets, e-games) was collected using the Adolescent Sedentary Activity Questionnaire (Hardy et al., 2007).

**Table 1:** Summary of Australian health behavior recommendations for children and adolescents.

Health indicator	Measurement tool and source	Recommendation	Reference
Daily serves of fruit^a^	Questionnaire (Flood et al., 2014)	Children age 4–8 years (years K, 2 and 4) consume ≥ 1½ serves daily; Children age 9–18 years (years 6, 8 and 10) consume ≥ 2 serves daily.	(National Health and Medical Research Council, 2013)
Daily serves of vegetables^b^	Questionnaire (Flood et al., 2014)	Children age 4–8 years (years K and 2) ≥ 4½ serves daily; Children age 9–11 years (years 4 and 6) consume ≥ 5 serves daily; Boys age 12–16 years (years 8 and 10) consume ≥ 5½ serves; girls 5 serves daily	(National Health and Medical Research Council, 2013)
Daily PA	Questionnaire (Prochaska et al., 2001)	Children age 5–18 years ≥ 60 min daily	(Department of Health, 2014a, Department of Health, 2014b)
Sleep	Questionnaire (Wolfson et al., 2003)	Children age 6–13 years; 9–11 h/night; Adolescents age 13–18 years; 8–10 h/night	(Wolfson et al., 2003)
Screen-time	Questionnaire (Hardy et al., 2007)	Children age 5–18 years to limit screen-time to < 2 h/day	(Department of Health, 2014a, Department of Health, 2014b)
Dental (tooth brushing)	Questionnaire (Harford and Luzzi, 2013)	Brush teeth ≥ 2 times/day	(Centre for Oral Health Strategy NSW, 2014)

a The response options for this question were integers hence analysis was based on 2-serves/day.

b The response options for this question were integers hence analysis was based on 4 or 5-serves/day.

Awareness of the PA and screen-time recommendations was assessed using the following questions; *How many minutes of PA is recommended that young people do each day?* and *Up to how many hours of TV, video, DVD or computer games is it recommended that young people watch each day?* Correct responses comprised accurate times (i.e., 60-minutes for PA and 2-hours for screen-time), all other responses including ‘Don't know’ were considered as not knowing the recommendations. We did not collect information about awareness of recommendations for fruit, vegetables, sleep, and oral hygiene.

Sociodemographic information included the child's sex, date of birth, language spoken most often at home, and postcode of residence. The Australian Bureau of Statistics' Socioeconomic Index for Areas Index of Relative Socioeconomic Disadvantage was derived at the postcode level as a proxy measure for neighborhood socioeconomic status (SES) (Australian Bureau of Statistics, 2013a) and used to rank children's postcodes into low, middle, and high tertiles of neighborhood SES. Postcode of residence was also used to determine residential locality (urban and rural) (Australian Bureau of Statistics, 2013b). Language spoken most often at home was used to categorize children as English speaking or non-English speaking (Australian Bureau of Statistics, 2011).

### 2.2 Statistical analysis

All analyses were conducted using Complex Samples SPSS (version 22 for Windows, IBM Corporation, Chicago, IL, USA) to account for the cluster design of the study and to adjust for the standard errors and 95% confidence intervals. Post-stratification weights were calculated to permit inferences from children included in the sample to the populations from which they were drawn, and to have the tabulations reflect estimates of the population totals. Children were stratified according to respondent; the children's group comprised students in kindergarten and years 2 and 4 (parent-report) and the adolescent group comprised students in years 6, 8 and 10 (self-report).

First, we compared the sociodemographic characteristics and adherence of health behavior recommendation between children and adolescents using chi-square tests. Then, for each health behavior recommendation, we performed logistic regression to examine the differences in the proportion of adherence by sex, residence, SES, and language spoken at home, mutually adjusted for other sociodemographic variables in the model. We present the adjusted odds ratios and their corresponding 95% confidence intervals for each independent variable. Overall adherence was calculated as the sum of meeting recommendations and assessed by sociodemographic characteristics. The significance level was set at 0.05.

## 3 Results

Of the 7555 children who participated in the survey, 52% were girls, most were from English-speaking backgrounds (87%), lived in urban areas (76%), and 24%, 34% and 42% were from low, middle, and high SES neighborhoods, respectively. There were no significant differences in sociodemographic characteristics of children and adolescents (Table 2).

**Table 2:** Socio-demographic characteristics of the children (n = 7555) in Australia, 2015.

Characteristic	Children	Adolescents	P-value^a^
n	3884	3671	
Mean age (years, SD)	7.5 (1.7)	13.2 (1.7)	< 0.001
Girls (%)	51.0 (48.4, 53.7)	49.6 (43.7, 55.6)	0.66
Home residence (%)			
Urban	78.6 (62.8, 88.9)	73.9 (62.8, 82.6)	0.48
Rural	21.4 (11.1, 37.2)	26.1 (17.4, 37.2)	
Socio-economic background (%)			
Low	21.8 (12.3, 35.5)	31.3 (23.3, 40.7)	0.21
Middle	33.6 (21.8, 47.9)	33.2 (25.7, 41.7)	
High	44.6 (30.9, 59.2)	35.4 (26.8, 45.2)	
Cultural background (%)			
English-speaking	87.2 (81.3, 91.4)	87.5 (82.9, 91.0)	0.91
Non-English speaking	12.8 (8.6, 18.7)	12.5 (9.0, 17.1)	

a Weighted data.

Adherence to each recommendation is presented in Table 3, stratified by age group. Overall, the prevalence of adhering to health behavior recommendations was low, and there were significant differences between children and adolescents. Adherence rates for children and adolescents were particularly low for vegetables (2.8% and 11.1%, respectively), PA (25.1% and 12.9%, respectively), and screen-time (15.2% and 19.7%, respectively). Almost two thirds of children (65.7%) and less than half of adolescents (46.2%) met sleep recommendations, less than two thirds of children (63.6%), seven in ten adolescents (70.2%) met the dental recommendation to brush teeth twice a day, and almost four in five children (76.8%) and adolescents (80.5%) met the fruit recommendation.

**Table 3:** Prevalence of adherence to health behavior recommendations, by age group in Australia, 2015.

Recommendation	Children	Adolescents	P-value^a^
Fruit and vegetables (diet) (%)			
Fruit serves	76.8 (74.3, 79.1)	80.5 (78.1, 82.7)	**0.019**
Vegetable serves	2.8 (2.1, 3.6)	11.1 (9.6, 12.7)	<** 0.001**
Fruit and vegetables combined	2.4 (1.8, 3.1)	9.8 (8.4, 11.3)	<** 0.001**
PA (%)			
Daily PA	25.1 (22.7, 27.7)	12.9 (11.2, 14.7)	<** 0.001**
Sleep (%)			
Every night	65.7 (63.5, 67.9)	46.2 (44.0, 48.5)	<** 0.001**
School nights	75.2 (72.8, 77.4)	76.8 (75.1, 78.5)	0.26
Non-school nights	79.7 (77.8, 81.4)	57.1 (55.1, 59.0)	<** 0.001**
Dental (%)			
Brush teeth ≥ 2 day	63.6 (60.0, 67.1)	70.2 (67.5, 72.7)	**0.002**
Screen-time (%)			
Every day	15.2 (13.5, 17.0)	19.7 (17.5, 22.0)	<** 0.001**
Week days	61.4 (57.2, 65.5)	44.4 (41.0, 47.9)	<** 0.001**
Weekend days	16.2 (14.6, 17.9)	23.4 (21.2, 25.7)	<** 0.001**

Significant values are in bold.

a Based on chi-square tests.

Awareness of recommendations was low with less than one third (30.7%) of parents of children and 24% of adolescents correctly identified the PA recommendation and 15.1% of parents and 9.5% of adolescents correctly identified the screen-time recommendation. Children were more likely to adhere to the PA recommendation (OR 1.71, 95%CI: 1.39, 2.10) when parents were aware with the recommendation, whereas parents' awareness of the screen-time recommendation was not related to a child's adherence (P = 0.11). Adolescents' awareness of the PA recommendation was not associated with adherence to the recommendation (P = 0.11), but adolescents who were aware of screen-time recommendations were 60% less likely to adhere to the screen-time recommendation (OR 0.40, 95%CI: 0.27, 0.61). (Data not shown).

**Fig. 1:**
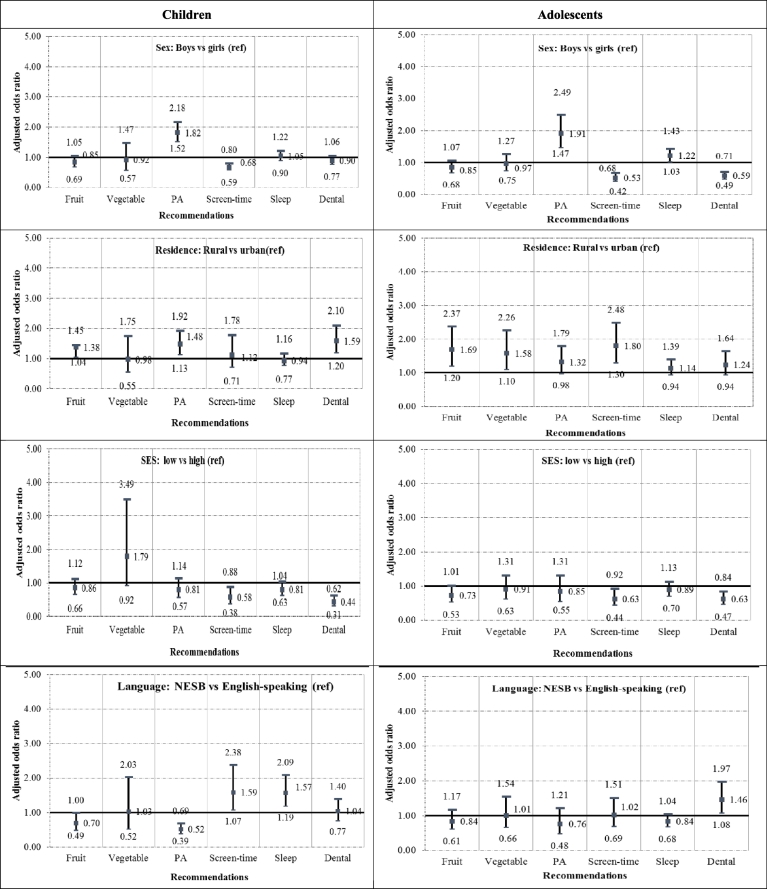
Adjusted odds ratio of adhering to recommendations, by socio-demographic characteristics and age group, in Australia, 2015. ^⁎^Adjusted for age, sex and other sociodemographic characteristics (residence, SES, language background). Models for sex were adjusted for age and sociodemographic characteristics. PA = PA; In SES models, children and adolescents from middle SES neighborhoods are excluded from the analysis.

Fig. 1 shows the adjusted odds ratio of adhering to recommendations by sociodemographic characteristics and age group. Compared with girls, boys from both age groups were more likely to adhere to recommendations for daily PA and less likely to adhere to the screen-time recommendation. Adolescent boys were more likely to adhere to sleep recommendations (AOR 1.22, 95%CI: 1.03, 1.43) and less likely to brush their teeth twice daily, (AOR 0.59, 95%CI: 0.49, 0.71), compared with adolescent girls. Children from rural areas were more likely to adhere to PA and dental recommendations, compared with children living in urban areas and adolescents from rural areas were more likely to adhere to fruit, vegetable and screen time recommendations compared with adolescents living in urban areas. Children and adolescents from low SES neighborhoods were less likely to adhere to screen-time and dental recommendations compared with peers from high SES neighborhoods. Compared with children from English-speaking backgrounds, children from NESB were less likely to adhere to the PA recommendation (AOR 0.52, 95%CI: 0.39, 0.69) but more likely to adhere to screen-time (AOR 1.59, 95% CI: 1.07, 2.38) and sleep (AOR 1.57, 95%CI: 1.19, 2.09) recommendations. Adolescents from NESB were more likely to brush their teeth twice daily, compared with adolescents from English-speaking backgrounds (AOR 1.46, 95%CI: 1.08, 1.97).

Table 4 shows the mean number and distribution of the number of recommendations that children and adolescents adhered to overall and by sociodemographic characteristics. Of the six recommendations examined, on average children adhered to 2.5 and adolescents 2.3 recommendations. Three percent and 4% of children and adolescents, respectively, adhered to none of the recommendations and < 0.3% adhered to all six recommendations. Adherence to recommendations was marginally significantly higher among children and adolescents from rural areas, compared with peers living in urban areas (children 2.7 vs 2.4, respectively; adolescents 2.6 vs 2.3, respectively) and among children and adolescents from high, compared with low SES neighborhoods (children 2.6 vs 2.2, respectively; adolescents 2.5 vs 2.2, respectively). The number of recommendations adhered to by children differed significantly according to SES and among adolescence marginal difference were observed according to residence and SES. Table 5 shows that adherence to ≤ 3 recommendations was higher among children and adolescents from urban, compared with rural areas, among adolescent boys, compared with girls, and among children from low, compared with high, SES neighborhoods.

**Table 4:** Distribution of number of recommendations adhered by socioeconomic characteristics and age group, in Australia, 2015.

Adherence to recommendations	All	Residence (%)		SES (%)^a^		Language background (%)	
Children		Urban	Rural	Low	High	English	Non-English speaking
Mean (n, SE)	*2.5* (*0.4*)	*2.4* (*0.3*)	***2.7*** (***0.7***)⁎	*2.2* (*0.5*)	***2.6*** (***0.5***)⁎	*2.5* (*0.3*)	*2.5* (*0.8*)
None (%)	3.3	2.2	2.6	3.6	1.5	2.2	2.8
One (%)	16.7	15.9	12.1	19.6	14.0	14.3	18.9
Two (%)	34.1	34.5	33.5	39.1	31.5	34.1	35.1
Three (%)	30.8	32.7	33.1	25.2	36.6	33.4	29.6
Four (%)	12.5	12.4	15.9	10.8	13.8	13.5	11.0
Five (%)	2.3	2.3	2.5	1.3	2.5	2.4	2.5
Six (%)	0.2	0.0	0.3	0.2	0	0.1	0.1
P-value		0.145		<** 0.001**		0.37	

Adolescents							
Mean (n)	*2.3* (*0.4*)	*2.3* (*0.3*)	***2.6*** (***0.6***)⁎	*2.2* (*0.6*)	***2.5*** (***0.6***)⁎	*2.3* (*0.3*)	*2.3* (*0.6*)
None (%)	4.4	4.7	3.5	5.0	3.6	4.2	5.2
One (%)	18.4	19.1	16.5	22.9	15.4	17.9	21.2
Two (%)	34.0	34.4	32.8	32.7	33.4	33.9	33.7
Three (%)	28.8	28.9	28.7	26.2	32.4	28.9	28.8
Four (%)	11.9	11.0	14.6	11.0	12.9	12.4	10.2
Five (%)	2.2	1.7	3.7	2.0	2.0	2.3	0.9
Six (%)	0.3	0.3	0.3	0.	0.3	0.4	0
P-value		**0.045**		**0.047**		0.135	

Significant values are in bold. Italicized values are means(SD).

⁎ P < 0.001 based on chi square tests; SES = socioeconomic status.

a Children and adolescents from middle SES neighborhoods are excluded from the analysis.

**Table 5:** Adjusted odds ratio of adhering to ≤ 3 recommendations, by socio-demographic characteristics and child group, in Australia, 2015⁎.

	Children	Adolescents
Adherence to ≤ 3 recommendations (%)	84.3	85.5
Sex		
Girls (ref)	1.0	1.0
Boys	0.88 (0.72, 1.07)	**1.32** (**1.02**, **1.70**)
Residence		
Rural (ref)	1.0	1.0
Urban	**1.56** (**1.06**, **2.31**)	**1.84** (**1.34**, **2.52**)
Socioeconomic		
High (ref)	1.0	1.0
Medium	1.16 (0.83, 1.63)	1.30 (0.96, 1.75)
Low	**1.63** (**1.10**, **2.41**)	1.42 (0.93, 2.16)
Language background		
English-speaking (ref)	1.0	1.0
Non-English speaking	1.01 (0.68, 1.50)	1.12 (0.78, 1.60)

Significant values are in bold.

⁎ Each model is mutually adjusted for sex, age, residence, SES, and language background.

## 4 Discussion

Based on a representative population health survey of children age 5–16 years we estimate that adherence to health behavior recommendations ranged from 7% (vegetables) to 79% (fruit) with significant differences in adherence rates according to age, residence, SES neighborhood, and language background. Information on adherence to health behavior recommendations by demographic characteristics is useful to determine where best to invest effort and resources to improve healthy lifestyle adherence.

There is consistent evidence that premature morbidity and mortality associated with NCDs including cardiovascular disease, stroke, diabetes, osteoporosis and various cancers can be prevented through engaging in healthy lifestyle behaviors (World Health Organization, 2004). Efforts to promote adherence to lifestyle health behavior recommendations during childhood is considered the most cost effective and feasible approach to reduce NCDs in adulthood (World Health Organisation, 2016). In children, poor adherence to health behavior recommendations has been associated with obesity, a precursor for many NCDs. Importantly, a number of studies show that children who adhere to multiple health behavior recommendations have a lower odds of obesity (Roman-Viñas et al., 2016, Pérez-Rodrigo et al., 2016, Wijnhoven et al., 2015, Santaliestra-Pasias et al., 2015), which may suggest each health behavior independently contributes to health and interventions should promote adherence to dietary, physical activity, sleep and screen-time recommendations simultaneously.

The health behavior recommendations we examined are based on evidence that adherence reduces children's risk of NCDs. We deliberately did not present adherence by weight status because overall adherence rates were low across all children, not only those in unhealthy weight categories. While inter-study comparisons on children's adherence to national health recommendations are difficult because of different survey methodologies, including differences in measurement methods and recommendation prescriptions, our findings are analogous to other population-based surveys in Australia (Bell et al., 2016, Australian Bureau of Statistics, 2013c, Australian Bureau of Statistics, 2013d) and internationally (Roman-Viñas et al., 2016, Tremblay et al., 2014, Santaliestra-Pasías et al., 2014, Fakhouri et al., 2013) that also indicate children's adherence to multiple health behavior recommendations are sub-optimal.

Of all six recommendations we examined, children and adolescents adhered to 2.5 and 2.3, respectively. Only 3% of children and adolescents in this study met zero recommendations, which is lower than similar studies of children's adherence to health behavior recommendations. In Canada, 83% of children do not meet sleep, PA and screen-time recommendations (Carson et al., 2017), and in American, 19% and 33% of children and adolescents do not meet sleep, PA, screen-time and dietary recommendations (Haughton et al., 2016). Evidence from previous research shows that the more health behavior recommendations children and adolescents met, the better their overall health (Roman-Viñas et al., 2016). Awareness of recommendations may be a contributing factor to adherence. In this study, children whose parents were aware of the PA recommendation were more likely to meet the recommendation however adolescents aware of the screen-time recommendation were more likely to exceed the recommendation. This finding suggests that qualitative research is required to understand how recommendations are interpreted in the community which can inform communication of the importance of adherence.

Dietary recommendations are based on the strong evidence that a healthy diet protects against malnutrition (underweight and overweight) and prevent NCDs (World Health Organization, 2004). Specific recommendations are developed for fruit and vegetables because they are an important source of nutrients, phytochemicals, dietary fiber and high daily intakes are associated with good health (Boeing et al., 2012). The promotion of fruit and vegetable consumption is key global strategy to minimize risk of NCDs (World Health Organization, 2004). Although jurisdictions differ slightly in their recommendations, a consistent finding is children's adherence to daily intake of vegetables is low, and is much lower than adherence for fruit intakes (Lynch et al., 2014). In this survey, 79% of children and adolescent's met the fruit recommendation and only 7% the recommendation for vegetables. With the exception of adolescents from rural areas, there were no consistent demographic differences, indicating population-wide programs to improve daily fruit and vegetable intake are justified. Government-funded, school-based fruit and vegetable programs have been implemented in many countries over many years with varying degrees of success (Ganann et al., 2014), which suggests that other strategies such as discounting fruit and vegetables (Waterlander et al., 2013) may add value to current interventions to increase adherence to these dietary recommendations.

There is consistent evidence that daily PA bestows beneficial effects on musculoskeletal health and fitness, cardiovascular health, adiposity, mental health, lipids and cardiovascular risk factors (Strong et al., 2005, Janssen and LeBlanc, 2010). One-in-five (20%) children and adolescents in this study adhered to the PA recommendation with boys (compared with girls) and children living in rural, compared with urban areas more likely to adhere and children from NESB, less likely, compared with children from English-speaking backgrounds. Overall, our findings are comparable to many but not all jurisdictions (Tremblay et al., 2016). In countries with poor PA infrastructure children had higher PA and lower sedentariness, while children in countries with better infrastructure had lower PA and higher sedentariness. It has been suggested that factors such as children's autonomy to play, active travel, or chore requirements and fewer attractive sedentary pursuits, rather than infrastructure and structured activities, may facilitate higher levels of PA in children (Tremblay et al., 2016). This finding suggests that focusing only on the physical environment and development of PA policies is not sufficient to improve children's adherence to the recommendation. Innovative strategies may be required to encourage children to switch from sedentary to physically active pursuits.

Sedentariness, or sitting, is associated with increased risk of cardio-metabolic disease, all-cause mortality and a variety of physiological and psychological problems; conversely any reduction in children's time spent sitting is associated with lower NCDs health risks (Saunders et al., 2014). Screen-time (i.e. TV, computers, smart phones, e-devices) is the primary contributor to the total time spent in sedentary behaviors among young people (Biddle et al., 2014), and longer screen-time in children is associated with detrimental health effects including reduced physical and psychosocial health (Tremblay et al., 2011), emotional problems and poorer well-being (Hinkley et al., 2014). Less than one in five children and adolescents (18%) in this study adhered to the recommendation, with boys and children and adolescents from low SES neighborhoods less likely to meet the recommendation, which is comparable to other jurisdictions (Tremblay et al., 2014). The innovation, proliferation and ubiquitous presence of screen devices in daily life has led to discussions about the relevance of the 20-year old recommendation to limit screen-time to < 2-hours/day in contemporary society. Although the evidence shows excessive screen-time can have deleterious effects, the US has revised their recommendations, removing the 2-hour prescription for 5–18 year olds (Hill et al., 2016). The impact of revising screen-time recommendations on children and adolescents' health is yet to be determined and needs close monitoring given the current low PA and high sitting time prevalence.

Inadequate and poor quality and quantity of sleep in children has been associated with cardiometabolic risk (Narang et al., 2012), behavioral problems, including aggression and attention-deficit/hyperactivity disorder, poor sociability, learning disabilities, and obesity (Stein et al., 2001). More than half (56%) of children and adolescents met the recommendation in this study however children were more likely to meet the recommendation compared with adolescents. Our estimates are higher than those for children and adolescents in Canada, where 24% meet sleep recommendations (Carson et al., 2017), and in the US where 60% of adolescents sleep less than eight hours on school nights (National Sleep Foundation, 2011). A growing area of concern is the use of screen devices before bed-time and the impact this practice has on sleep hygiene. In the US, 72% of adolescents used their cell phones in their bedroom the hour before bedtime which was associated to insufficient sleep duration and poor sleep quality (Gradisar et al., 2013). This latter finding suggests that while establishing regular bed-times is one strategy to improve children's sleep hygiene it may not be sufficient if there are no limits on screen devices in bedrooms, particularly at bed-time.

Good dental health enables an individual to eat, speak and socialize without active disease, discomfort or embarrassment, which in turn contributes to general well-being (Moynihan, 2005). Globally, dental caries are the most commonly occurring oral disease in children (Selwitz et al., 2007) and population preventive measures are to brush teeth twice a day (for at least 2 min with fluoride toothpaste) (The Royal Australasian College of Physicians, 2012). The majority of children and adolescents in this study met the recommendation, but one third (33%) did not. Children and adolescents from low SES neighborhoods, compared with peers from high SES neighborhoods and adolescent boys, compared with adolescent girls were less likely to adhere to the recommendation. Global comparisons are difficult as the estimates reported by the Health Behavior in School-aged Children study are based on brushing more-than-once-day, but as with this study, show social disparities in teeth brushing behavior (Currie et al., 2012).

The strength of this study is the use of a representative population children health survey to estimate adherence to six important health behavior recommendations that reduce risk of NCD's in children. The use of proxy- and self-report to measure the six health behaviors and determine the proportion of children adhering to those recommendations is recognized as a limitation. Additionally, we only asked awareness of two of the six recommendations, so we are unable to determine overall awareness of health behavior recommendations which would be useful for health promotion efforts to increase children's adherence.

## 5 Conclusion

The proportion of children and adolescents meeting government health behavior recommendations is sub-optimal, with < 1% meeting all six recommendations. Social disparities exist, with children from low SES neighborhoods and urban areas meet fewer recommendations compared with children from high SES neighborhoods and rural areas, indicating efforts to improve adherence need further work in these population groups. The recommendations we examined are designed to reduce children's risk of future NCDs and the overall low adherence is a potential warning that the incidence of NCDs among the current generation of children may increase leading to greater health and economic burdens on communities. Awareness of the recommendations (PA and screen-time) was low which is of concern. Ironically, the heavy use of social media among children and adolescents may be a useful strategy to promote recommendations by embedding them in related advertisements.

## Conflict of interest

The authors have no conflicts of interest relevant to this article to disclose.

## References

[bb0005] Australian Bureau of Statistics (2011). Australian Standard Classification of Languages (ASCL) 2nd Edition, 2011. 1267.0 Ed.

[bb0010] Australian Bureau of Statistics (2013). Census of Population and Housing: Socio-Economic Indexes for Areas (SEIFA), Australia—Data Only, 2011. Catno2033055001—[Internet]. http://www.abs.gov.au/AUSSTATS/abs@.nsf/DetailsPage/2033.0.55.0012006?OpenDocument.

[bb0015] Australian Bureau of Statistics (2013). Australian Statistical Geography Standard (ASGS)—Remoteness Structure. Canberra: 1270.0.55.005.

[bb0020] Australian Bureau of Statistics (2013). Australian Health Survey: Updated Results, 2011–2012.

[bb0025] Australian Bureau of Statistics (2013). Australian Health Survey: Physical Activity, 2011–12.

[bb0030] Bell L., Ullah S., Olds T. (2016). Prevalence and socio-economic distribution of eating, physical activity and sedentary behaviour among South Australian children in urban and rural communities: baseline findings from the OPAL evaluation. Public Health.

[bb0035] Biddle S.J., Petrolini I., Pearson N. (2014). Interventions designed to reduce sedentary behaviours in young people: a review of reviews. Br. J. Sports Med..

[bb0040] Boeing H., Bechthold A., Bub A. (2012). Critical review: vegetables and fruit in the prevention of chronic diseases. Eur. J. Nutr..

[bb0045] Carson V., Chaput J.-P., Janssen I. (2017). Health associations with meeting new 24-hour movement guidelines for Canadian children and youth. Prev. Med..

[bb9005] Centre for Oral Health Strategy NSW (2014). The Early Childhood Oral Health Guidelines for Child Health Professionals.

[bb0050] Currie C., Zanotti C., Morgan A. (2012). Social Determinants of Health and Well-Being among Young People.

[bb6000] Department of Health (2014). Australia's Physical Activity and Sedentary Behaviour Guidelines for Children (5-12 years).

[bb6005] Department of Health (2014). Australia's Physical Activity and Sedentary Behaviour Guidelines for Children (13-17 years).

[bb0055] Ding D., Rogers K., van der Ploeg H. (2015). Traditional and emerging lifestyle risk behaviors and all-cause mortality in middle-aged and older adults: evidence from a large population-based Australian cohort. PLoS Med..

[bb0060] Fakhouri T.H., Hughes J.P., Brody D.J. (2013). Physical activity and screen-time viewing among elementary school-aged children in the United States from 2009 to 2010. JAMA Pediatr..

[bb0065] Flood V., Webb K., Rangan A.M. (2005). Recommendations for Short Questions to Assess Food Consumption in Children for the NSW Health Surveys.

[bb6010] Flood V.M., Wen L.M., Hardy L.L. (2014). Reliability and validity of a short FFQ for assessing the dietary habits of 2-5-year-old children, Sydney, Australia. Public Health Nutr..

[bb0070] Forouzanfar M.H., Afshin A., Alexander L.T. (2015). Global, regional, and national comparative risk assessment of 79 behavioural, environmental and occupational, and metabolic risks or clusters of risks, 1990–2013: a systematic analysis for the Global Burden of Disease Study. Lancet.

[bb0075] Ganann R., Fitzpatrick-Lewis D., Ciliska D. (2014). Enhancing nutritional environments through access to fruit and vegetables in schools and homes among children and youth: a systematic review. BMC. Res. Notes.

[bb0080] Gradisar M., Wolfson A.R., Harvey A.G. (2013). The sleep and technology use of Americans: findings from the National Sleep Foundation's 2011 sleep in America poll. J. Clin. Sleep Med..

[bb0085] Hardy L.L., Booth M.L., Okely A.D. (2007). The reliability of the Adolescent Sedentary Activity Questionnaire (ASAQ). Prev. Med..

[bb0090] Hardy L.L., Mihrshahi S., Drayton B.A. (2017). NSW Schools Physical Activity and Nutrition Survey (SPANS) 2015 Full Report.

[bb6050] Harford J., Luzzi L. (2013). Child and teenager oral health and dental visiting: Results from the National Dental Telephone Interview Survey 2010. Dental Statistics and Research Series no. 64.

[bb0095] Haughton C.F., Wang M.L., Lemon S.C. (2016). Racial/ethnic disparities in meeting 5-2-1-0 recommendations among children and adolescents in the United States. J. Pediatr..

[bb1000] Hill D., Ameenuddin N., Chassiakos Y.L.R., Cross C., Hutchinson J., Levine A., Boyd R., Mendelson R., Moreno M., Swanson W.S. (2016). Media and young minds. Pediatrics.

[bb0100] Hinkley T., Verbestel V., Ahrens W. (2014). Early childhood electronic media use as a predictor of poorer well-being: a prospective cohort study. JAMA Pediatr..

[bb0105] Hirshkowitz M., Whiton K., Albert S.M. (2015). National Sleep Foundation's sleep time duration recommendations: methodology and results summary. Sleep Health.

[bb0110] Janssen I., LeBlanc A.G. (2010). Systematic review of the health benefits of physical activity and fitness in school-aged children and youth. Int. J. Behav. Nutr. Phys. Act..

[bb0115] Kovacs E., Siani A., Konstabel K. (2014). Adherence to the obesity-related lifestyle intervention targets in the IDEFICS study. Int. J. Obes..

[bb0120] Lynch C., Kristjansdottir A.G., Te Velde S.J. (2014). Fruit and vegetable consumption in a sample of 11-year-old children in ten European countries—the PRO GREENS cross-sectional survey. Public Health Nutr..

[bb0130] Moynihan P.J. (2005). The role of diet and nutrition in the etiology and prevention of oral diseases. Bull. World Health Organ..

[bb0135] Narang I., Manlhiot C., Davies-Shaw J. (2012). Sleep disturbance and cardiovascular risk in adolescents. Can. Med. Assoc. J..

[bb9000] National Health and Medical Research Council (2013). Australian Dietary Guidelines.

[bb0140] National Sleep Foundation (2011). Sleep in America Poll: Communications Technology in the Bedroom.

[bb0145] Pérez-Rodrigo C., Gil Á., González-Gross M. (2016). Clustering of dietary patterns, lifestyles, and overweight among Spanish children and adolescents in the ANIBES study. Nutrients.

[bb0150] Prochaska J.J., Sallis J.F., Long B. (2001). A physical activity screening measure for use with adolescents in primary care. Arch. Pediatr. Adolesc. Med..

[bb0155] Roman-Viñas B., Chaput J.-P., Katzmarzyk P.T. (2016). Proportion of children meeting recommendations for 24-hour movement guidelines and associations with adiposity in a 12-country study. Int. J. Behav. Nutr. Phys. Act..

[bb0160] Santaliestra-Pasías A.M., Mouratidou T., Verbestel V. (2014). Physical activity and sedentary behaviour in European children: the IDEFICS study. Public Health Nutr..

[bb0165] Santaliestra-Pasias A.M., Mouratidou T., Reisch L. (2015). Clustering of lifestyle behaviours and relation to body composition in European children. The IDEFICS study. Eur. J. Clin. Nutr..

[bb0170] Saunders T.J., Chaput J.-P., Tremblay M.S. (2014). Sedentary behaviour as an emerging risk factor for cardiometabolic diseases in children and youth. Can. J. Diabetes.

[bb0175] Selwitz R.H., Ismail A.I., Pitts N.B. (2007). Dental caries. Lancet.

[bb0180] Stein M.A., Mendelsohn J., Obermeyer W.H. (2001). Sleep and behavior problems in school-aged children. Pediatrics.

[bb0185] Strong W.B., Malina R.M., Blimkie C.J. (2005). Evidence based physical activity for school-age youth. J. Pediatr..

[bb0190] Swinburn B.A., Sacks G., Hall K.D., McPherson K., Finegood D.T., Moodie M.L., Gortmaker S.L. (2011). The global obesity pandemic: shaped by global drivers and local environments. Lancet.

[bb0195] The Royal Australasian College of Physicians (2012). Royal Australasian College of Physicians: Oral Health of Children and Young People.

[bb0200] Tremblay M.S., LeBlanc A.G., Kho M.E. (2011). Systematic review of sedentary behaviour and health indicators in school-aged children and youth. Int. J. Behav. Nutr. Phys. Act..

[bb0205] Tremblay M.S., Gray C.E., Akinroye K. (2014). Physical activity of children: a global matrix of grades comparing 15 countries. J. Phys. Act. Health.

[bb0210] Tremblay M.S., Barnes J.D., Gonzalez S.A. (2016). Global matrix 2.0: report card grades on the physical activity of children and youth comparing 38 countries. J. Phys. Act. Health.

[bb0215] Waterlander W.E., de Boer M.R., Schuit A.J. (2013). Price discounts significantly enhance fruit and vegetable purchases when combined with nutrition education: a randomized controlled supermarket trial. Am. J. Clin. Nutr..

[bb0220] Wijnhoven T.M., van Raaij J.M., Yngve A. (2015). WHO European childhood obesity surveillance initiative: health-risk behaviours on nutrition and physical activity in 6–9-year-old schoolchildren. Public Health Nutr..

[bb0225] Wolfson A.R., Carskadon M.A., Acebo C. (2003). Evidence for the validity of a sleep habits survey for adolescents. Sleep.

[bb0230] World Health Organisation (2016). Report of the Commission on Ending Childhood Obesity.

[bb0235] World Health Organization (2004). Global Strategy on Diet, Physical Activity and Health.

